# How useful is ChatGPT in answering point-of-care questions in primary care?

**DOI:** 10.31744/einstein_journal/2025CE1749

**Published:** 2025-10-03

**Authors:** Filipe Prazeres

**Affiliations:** 1 Universidade da Beira Interior Faculdade de Ciências da Saúde Covilhã Portugal Faculdade de Ciências da Saúde, Universidade da Beira Interior, Covilhã, Portugal.; 2 USF Beira Ria Gafanha da Nazaré Portugal USF Beira Ria, Gafanha da Nazaré, Portugal.; 3 Universidade do Porto Faculdade de Medicina Departamento de Medicina da Comunidade, Informação e Decisão em Saúde Porto Portugal RISE-Health, Departamento de Medicina da Comunidade, Informação e Decisão em Saúde, Faculdade de Medicina, Universidade do Porto, Porto, Portugal.

Dear Editor,

Physicians often encounter clinical questions during patient care.^(1)^ They often spend more than half an hour trying to find answers to these point-of-care questions, yet inquiries are not always resolved.^(2)^ For family physicians, electronic medical databases have shown promise in answering most clinical questions. However, point-of-care searches are not fast enough;^(3)^ slow internet connections and poor navigability limit the practical use of these resources during patient visits.^(4)^

"We can define our future by embracing artificial intelligence and using it to preserve our most precious resource – time with patients".^(5)^ Indeed, artificial intelligence has been embraced by millions of people, with ChatGPT alone reaching 200 million active weekly users worldwide.^(6)^ In medical education, ChatGPT can provide personalized and efficient learning for doctors, helping them strengthen their clinical reasoning and decision-making skills for better case analyses and diagnoses.^(7)^

The aim of the present study was to investigate the usefulness of ChatGPT-4o in answering point-of-care questions in primary care. In total, 200 clinical primary care questions were gathered from an openly available dataset deposited in the Zenodo repository in 2022^(8)^ (available at the following doi: 10.5281/zenodo.7275128). The data collection methods for the dataset have been previously described and published elsewhere.^(9)^ The choice to reuse questions from this dataset arose from their origins in real consultations between family physicians and patients. These questions capture authentic and common medical inquiries encountered in practice, directly reflecting the types of questions that arise at the point of care. All 200 selected questions have international relevance; questions specific to the collection region were excluded. Of these, 127 were background questions, 72 were foreground questions (covering differential diagnosis, diagnostic accuracy, treatment, etiology/harm, prognosis, and patient experiences), and one was related to ethics.

The questions included in this study were in Portuguese. They were submitted to ChatGPT-4o in Portuguese, without editing, and the responses were obtained in Portuguese. Each ChatGPT response was individually assessed for sentence count, word count, clinical adequacy (by direct comparison with online evidence-based resources, such as BMJ Best Practice, DynaMed, or UpToDate), source citations, and response speed.

ChatGPT-4o provided answers to all questions within a few seconds, with an average of 32±17 sentences and 444±200 words per response. The clinical adequacy of the answers was good, and the explanations were free of false information. However, only seven questions (3.5%) included source citations that referenced scientific societies or associations (two background questions and two treatment questions), the World Health Organization (one background question), the National Institute for Health and Care Excellence (one background question), and expert consensus (one treatment question).

Qualitatively, ChatGPT provided a diverse range of responses, ranging from high-quality content that exactly matched evidence-based resources to less-definitive answers. In some instances, ChatGPT outlined clinical flowcharts and tables; in others, it recommended medical evaluation (*e.g.,* therapeutic regimens may vary according to local guidelines or manufacturer's instructions, and precise indications should be based on medical assessment). ChatGPT sometimes accounted for varying levels of patients’ awareness of risk factors or behaviors, suggesting a broader medical evaluation than the recommended evidence-based resources. In other cases, it adopted a more patient-centered approach by offering patient guidance that was absent from evidence-based resources. However, ChatGPT's responses to specific medical scenarios were generally less accurate than their responses to the background questions. For example, in one clinical case involving a female patient, ChatGPT referenced a broad panel of laboratory tests, including prostate-specific antigen (PSA) measurements, reflecting a mismatch between the patient's sex and selected tests.

It was also observed that ChatGPT's straightforward, unified interface creates an impression of genuine understanding, making each conversation feel like a dialogue with an informed colleague or physician, a level of engagement not achievable with traditional, evidence-based online searches.

This study demonstrated that, globally, ChatGPT-4o can quickly provide concise responses to clinical questions raised by family physicians at the point of care, regardless of whether the questions are background, foreground, or ethical. These findings are significant because family physicians can query ChatGPT in their native language, without needing specific search strategies, making information retrieval easier and potentially saving time compared to traditional searches through medical databases or extensive literature. Previous research has shown that consulting online clinical summaries, composed of evidence syntheses, typically takes a median search time of 3.5 minutes (range: 1–13.5 minutes),^(9)^ hich is significantly longer than ChatGPT's response time of just a few seconds.

Given ChatGPT's vast knowledge base and popularity, family physicians may assume that they are accessing high-quality and consistently updated content and gain confidence in receiving answers or guidance, even for complex clinical questions. However, as observed in this study, ChatGPT's responses varied in quality, and although generally clinically adequate, lacked verifiable sources. ChatGPT has the potential to serve as a comprehensive medical resource; however, this can only be achieved if it incorporates verifiable evidence-based clinical data. Otherwise, there is a risk of providing inaccurate or misleading information that can negatively affect patient care. In addition, doctors remain accountable for errors resulting from the use of artificial intelligence.^(10)^

This study has some limitations. Given that the clinical adequacy of ChatGPT's answers is a subjective outcome and that it was evaluated by a single researcher, some degree of subjective bias cannot be entirely ruled out. A quantitative measure of the response adequacy was not conducted because of the heterogeneity of the questions and ChatGPT's responses. Additionally, the clinical adequacy of ChatGPT's responses is likely to vary depending on the specific questions asked and may be influenced by changes in ChatGPT versions over time.

Chatbots are effective because they maintain an uninterrupted experience, thereby avoiding disruptions, such as redirecting to other sites or citing specific sources. However, if chatbots are to be used to answer clinical questions from family physicians at the point-of-care, should they not be held to a higher standard, one that includes pinpointing the exact training data that inform their responses? The future will certainly inform us about this.
